# Markers of Dermal Fibroblast Subpopulations for Viable Cell Isolation via Cell Sorting: A Comprehensive Review

**DOI:** 10.3390/cells13141206

**Published:** 2024-07-17

**Authors:** Krzysztof Łuszczyński, Marta Soszyńska, Michał Komorowski, Paulina Lewandowska, Robert Zdanowski, Anna Sobiepanek, Marek Brytan, Jacek Malejczyk, Anna Lutyńska, Aneta Ścieżyńska

**Affiliations:** 1 Laboratory of Molecular Oncology and Innovative Therapies, Military Institute of Medicine National Research Institute, 128 Szaserów Street, 04-141 Warsaw, Poland; kluszczynski@wim.mil.pl (K.Ł.); rzdanowski@wim.mil.pl (R.Z.); alutynska@wim.mil.pl (A.L.); 2 Department of Histology and Embryology, Medical University of Warsaw, 02-004 Warsaw, Poland; marta.soszynska@wum.edu.pl (M.S.); michal.komorowski@wum.edu.pl (M.K.); plewandowska111@gmail.com (P.L.); jacek.malejczyk@wum.edu.pl (J.M.); 3 Chair of Drug and Cosmetics Biotechnology, Faculty of Chemistry, Warsaw University of Technology, 00-664 Warsaw, Poland; anna.sobiepanek@pw.edu.pl; 4 Department of Pharmacology and Toxicology, Military Institute of Hygiene and Epidemiology, 01-163 Warsaw, Poland; marek.brytan@wihe.pl

**Keywords:** fibroblast subpopulations, surface proteins, surface markers, skin, fluorescence-activated cell sorting, FACS, single-cell next-generation sequencing, scNGS, viable cell isolation

## Abstract

Fibroblasts are among the most abundant cell types in the human body, playing crucial roles in numerous physiological processes, including the structural maintenance of the dermis, production of extracellular matrix components, and mediation of inflammatory responses. Despite their importance, fibroblasts remain one of the least characterized cell populations. The advent of single-cell analysis techniques, particularly single-cell RNA sequencing (scRNA-seq) and fluorescence-activated cell sorting (FACS), has enabled detailed investigations into fibroblast biology. In this study, we present an extensive analysis of fibroblast surface markers suitable for cell sorting and subsequent functional studies. We reviewed over three thousand research articles describing fibroblast populations and their markers, characterizing and comparing subtypes based on their surface markers, as well as their intra- and extracellular proteins. Our detailed analysis identified a variety of distinct fibroblast subpopulations, each with unique markers, characteristics dependent on their location, and the physiological or pathophysiological environment. These findings underscore the diversity of fibroblasts as a cellular population and could lead to the development of novel diagnostic and therapeutic tools.

## 1. Introduction

Fibroblasts, along with epithelial and blood cells, make up one of the most common cell types in the human body [1]. Fibroblasts are the most frequent cell type of connective tissues throughout the human body and are responsible for a variety of functions such as structural maintenance of the dermis, production of extracellular matrix components, and mediation of inflammatory processes, as well as cellular proliferation and differentiation in response to chronic tissue damage and inflammation [2]. They detect infections and other danger signals, triggering host defense systems such as antimicrobial activity and leukocyte recruitment [3]. Furthermore, they are easily accessible and reprogrammable into induced pluripotent stem cells, making them a promising target for biological therapies [4]. Fibroblasts might also be a useful model for skin cytotoxicity testing to predict the surfactant potential for skin irritation or general mitochondrial-induced toxicity [5,6].

Fibroblasts are primarily located in the dermis of the human skin, the outermost protective barrier of the organism, which is divided into two layers: the upper, thin papillary layer just under the epidermis, and the lower, reticular layer, which is adjacent to the hypodermis [7]. Interestingly, fibroblast cells exhibit phenotypic heterogeneity among different tissues and even within the same tissue. Fibroblasts obtained from different anatomical sites might show distinct gene expression patterns [8]. Also, environmental factors such as UV radiation and pollution may influence fibroblast behavior. Exposure to these external triggers can result in oxidative stress, DNA damage, and changes in fibroblast signaling pathways [9,10].

Under normal conditions, fibroblasts regulate extracellular matrix turnover to preserve tissue homeostasis [11]. In injured skin, fibroblasts in the reticular layer mediate the initial steps of dermal healing, while those in the top layer of the dermis are recruited only after re-epithelialization [12]. When tissues are damaged, surrounding fibroblasts develop into myofibroblasts, which are highly contractile cells that generate a large amount of extracellular matrix proteins [13]. Excessive accumulation of ECM components in the dermis can lead to fibrotic skin diseases such as scleroderma [14], hypertrophic scars [15], and keloids [16]. Despite their importance, the fibroblast cell population is still one of the least characterized and has long been underappreciated.

Using single-cell RNA sequencing (scRNA-seq), thousands of individual cell transcriptomes have been identified so far, and numerous intracellular genes for different fibroblast lineages have been identified [17]. Although various research groups have been investigating transcriptome diversity in human skin cells, the results are limited and inconclusive because fibroblast subpopulations vary in size, composition among datasets, and expressed markers [18]. Nonetheless, studies have revealed previously unknown heterogeneity in normal human dermal fibroblasts, as well as disease-specific fibroblast subpopulations in a variety of skin diseases, such as fibrosis [19], atopic dermatitis [20], systemic sclerosis [21], psoriasis [22], and vitiligo [23].

Unfortunately, scRNA-seq research primarily focuses on identifying novel fibroblast subpopulations, while additional investigations focusing on isolated, viable, lineage-specific cells selected during fluorescence-activated cell sorting (FACS) are rarely performed. This might be due to a lack of antibodies that recognize cell membrane markers, as no definitive, distinct fibroblast-defining cell surface profile has been discovered thus far [24]. As a result, numerous studies have concentrated entirely on unsorted fibroblasts from a single tissue. However, the identification of different human fibroblast lineages is critical for expanding our understanding of dermal homeostasis and cellular function at the transcriptomic level, as well as evaluating fibroblasts’ unique properties, regulating their fibrogenic activity, and developing new treatment options.

Therefore, here, we provide, for the first time, a comprehensive review that includes a thorough analysis of over 3000 papers, revealing surface markers across different fibroblast populations that can be employed for cell sorting of living cells for future functional research.

## 2. Materials and Methods

Selected literature (n = 3048) was retrieved from the National Center for Biotechnology Information Database (https://www.ncbi.nlm.nih.gov/ accessed on 13 December 2023 and revisited on 12 March 2024). Search terms were “Humans”; “Fibroblasts”, “Population”, “Sorting”, “Surface Marker”, “Heterogeneity”, “Discrimination”, “Single Cell”, “FACS”, “Subpopulation”, “Subtype”, “Phenotypes”, “Dermatology”, “Skin”, “CD”, “Surface”, “Fibroblasts Phenotypes”, “Fibroblasts Heterogeneity”, “Aging”, “Exposure”, “UV” and “Pollution” combined in various modifications with Boolean operators “AND” and “OR”. Additional information was obtained by hand searches of relevant articles (n = 33) not identified in the PubMed database, as well as from the studied publications’ references. Studies involving cell cultures and/or animals were omitted from the analysis. A literature review was conducted from January to June 2024.

## 3. Results

**Figure 1:**
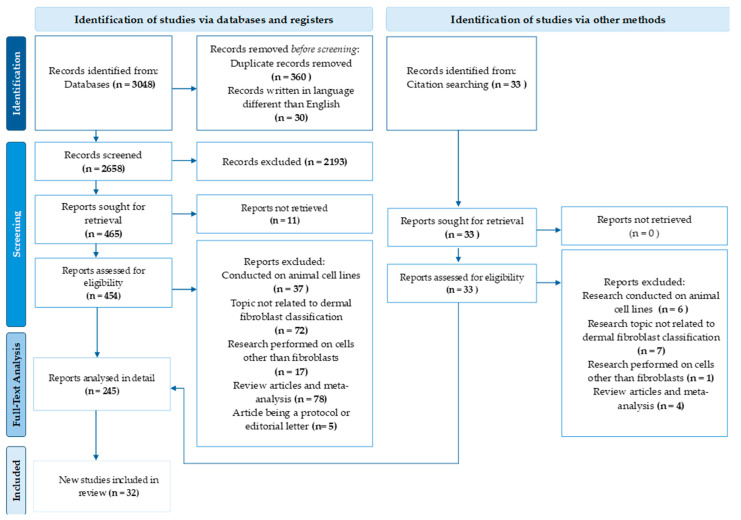
Flowchart of the selection process of the analyzed publications.

A flowchart of the search of the literature from the last 10 years is presented in Figure 1. Initially, over 3000 papers were selected for analysis. After final selection, papers that did not meet the inclusion criteria—such as those not in English, studies performed on cell lines and/or animals—were excluded. Consequently, 454 papers were assessed for eligibility and screened. From these, publications not relevant to the subject were excluded, resulting in 32 papers being selected for the final analysis.

The results were categorized into those obtained using single-cell sequencing methods (Table 1, Table 2, Table 3, Table 4 and Table 5) and those obtained through fluorescence-activated cell sorting (FACS) (Table 6).

In Table 1, the results from single-cell sequencing analyses of fibroblast subpopulations in normal and keloid skin tissues are presented. The reviewed studies identified genes encoding 12 distinct surface markers, namely ADAM Metallopeptidase Domain 12 (ADAM12), adenomatosis polyposis coli down-regulated 1 (APCDD1), platelet-derived growth factor receptor A (PDGFA, also known as CD140a), cluster of differentiation 26 or dipeptidyl peptidase-4 (CD26, also known as DPP4), Tumor Necrosis Factor Receptor superfamily member 12A (TNFR12A, also known as CD266), hematopoietic progenitor cell antigen CD34 (CD34), Complement Decay-Accelerating Factor (CD55), Thy-1 Membrane Glycoprotein (CD90), Glypican Proteoglycan 3 (GPC3), Syndecan-1 (SDC1), and Tetraspanin-8 (TSPAN8). Additionally, the studies revealed genes encoding approximately 40 unique non-surface markers, including 8 intracellular proteins (marked in red), 20 extracellular matrix proteins (marked in green), and 12 secreted proteins (marked in blue).

In Table 2 and Table 3, single-cell analyses performed on inflammatory and autoimmune skin diseases, such as atopic dermatitis, psoriasis, prurigo nodularis, lupus erythematosus, hidradenitis suppurativa, and vitiligo, are described. In these studies, genes encoding seven novel surface proteins were identified: Cluster of Differentiation 74 (CD74), Ectodermal Dysplasia Receptor Associated (EDRNA), Vascular Cell Adhesion Molecule 1 (VCAM1), Interferon-Induced Transmembrane Protein 2 and 3 (IFITM2 and IFITM3), Dipeptidase 1 (DPEP1), Tumor Necrosis Factor Receptor 1 and 2 (TNFR1/TNFR2, also known as CD120a and CD120b), and Interleukin 17 Receptor A and C (IL17RA/IL17RC). Additionally, genes encoding 19 novel non-surface proteins were identified.

In Table 4, the results of single-cell sequencing studies of skin diseases involving dysplasia and malignancies, such as actinic keratosis, basal cell carcinoma, and squamous cell carcinoma, are summarized. In these studies, genes encoding 10 novel cell surface markers were identified, including C-X-C Motif Chemokine Receptor (CXCR4), Fibroblast Activation Protein (FAP), Major Histocompatibility Complex, Class II, DR Alpha (HLA-DRA), Interleukin 7 Receptor (IL7R), Leucine Rich Repeat-Containing 15 (LRRC15), Neurogenic Locus Notch Homolog Protein 3 (NOTCH3), Repulsive Guidance Molecule A (RGMA), Scavenger Receptor Class A Member 5 (SCARA5), and SLIT and NTRK-like Family, Member 6 (SLITRK6).

In Table 5, single-cell sequencing analyses of fibroblast subpopulations involved in the process of skin aging are described. The included studies showed a high similarity of the cellular composition of skin samples from young and elderly patients and highlighted a distinct subtype associated with young skin—Dermal Sheath Fibroblasts characterized by the expression of genes encoding Dipeptidase 1 (DPEP1), Tenomodulin (TNMD), and Glypican-3 (GPC3).

In Table 6, publications analyzing various subpopulations of skin fibroblasts using FACS or other antibody-based methods are described. The majority of research has been performed on healthy skin and is focused on the division of fibroblasts into two phenotypically distinct subpopulations—CD36 positive reticular fibroblasts and CD39 positive papillary fibroblasts. Most populations were assessed based on the expression of surface markers, CD26, CD90, CD36, CD39 and FAP, in various combinations; on the other hand, unique subpopulations expressing genes encoding TGF beta receptor 1 (TGFB1R) or Corticotropin-releasing factor receptor 1 (CRHR1) and Synaptogyrin-2 (SYNGR2) were also discovered. This table also includes negative surface markers, such as Leukocyte Common Antigen (CD45) for identifying hematopoietic cells (leukocytes), E-cadherin (CDH1) for identifying epithelial cells, VCAM-1 (CD106) for identifying activated endothelial cells, Glycophorin A (CD235) for identifying erythroid cells, and Integrin Alpha 6 (CD49f) for identifying basal epithelial cells, including stem cells in epithelial tissues.

## 4. Discussion

Our understanding of fibroblast functional heterogeneity lags behind that of inflammatory cell types due to the absence of unique markers and the limitations of techniques such as fluorescence-activated cell sorting (FACS) on enzymatically digested tissue. Fibroblasts are among the most challenging cell types to classify due to their diversity and adaptability. Even within the same tissue sample, skin fibroblasts exhibit variability, likely influenced by their microenvironmental position [47]. The superficial papillary dermis, which hosts a diverse population of fibroblasts, is believed to contain various subpopulations essential for maintaining homeostasis and responding to pathological conditions in the skin [45]. Consequently, despite various methods existing for analyzing fibroblasts, our knowledge of tissue fibroblast repertoires remains incomplete. This gap hinders the identification of genes highly expressed in both layers of the dermis and in all cultured fibroblasts, but undetectable in other cultured cell types. Thus, finding universal pan-fibroblast surface markers has been challenging. Identifying and isolating these fibroblast populations is crucial for understanding their roles in tissue healing, fibrosis, and malignancy, yet despite their importance, discovering accurate pan-markers for fibroblasts remains difficult. Current markers such as Thy-1 (CD90) [48], Vimentin [49], Fibroblast Activation Protein (FAP) [50], and PDGFR-α (Platelet-Derived Growth Factor Receptor Alpha) [51] are widely used but have limitations due to their expression in various cell types. For instance, Thy-1 is also found in mesenchymal stem cells and endothelial cells [52], Vimentin is prevalent in macrophages and certain epithelial cells [53], FAP is mostly expressed in activated fibroblasts and does not represent the entire fibroblast population, and PDGFR-α is not exclusive to fibroblasts [54]. These limitations underscore the need for more specific and universally applicable pan-fibroblast markers. While small leucine-rich proteoglycans such as LUM (Lumican) and DCN (Decorin) have been identified as secreted pan-fibroblast markers [42], they cannot be used to isolate viable fibroblasts during cell sorting.

There are different approaches to analyzing fibroblast subpopulations. Among various studies, the majority of them focused either on single-cell next-generation sequencing (scNGS), or fluorescence-activated cell sorting (FACS), even though those studies are very limited.

### 4.1. Fibroblast Markers Identified during Single-Cell Sequencing

In papers focusing on scNGS in normal skin, researchers focus mainly on fibroblasts’ secretory characteristics (Table 1, Table 2, Table 3, Table 4 and Table 5). Unfortunately, interpreting these data is time-consuming and difficult to re-analyze due to the large number of fibroblast subpopulations, various combinations of markers used in RNA-seq studies to locate clusters of skin fibroblasts, and a lack of descriptive information on those cells. In general, normal skin fibroblasts were divided into clusters of secretory papillary fibroblasts, secretory reticular fibroblasts, mesenchymal fibroblasts, and pro-inflammatory fibroblasts (Table 1).

Secretory papillary fibroblasts are the main source of cells needed for hair development. They are mainly found in the upper papillary dermis and are known to express genes encoding papillary markers linked to Wnt/β-catenin signaling as well as collagen or extracellular matrix formation such as collagens (COL6A5, COL18A1), Wnt inhibitory factor 1 (WIF1), adenomatosis polyposis coli down-regulated 1 protein (APCDD1), and prostaglandin-H2 D-isomerase (PTGDS) [55]. Indeed, in the papers of Solé-Boldo L. et al. [26], Riedl JA et al. [27], and Shim J et al. [16], the expression of some of these markers was confirmed. Unfortunately, only one of those—the cell surface lipid-binding protein adenomatosis polyposis coli down-regulated 1 (APCDD1)—may be utilized as a surface marker in the sorting of live secretory papillary fibroblast cells. APCDD1 is an inhibitor of the Wnt signaling pathway that is abundantly expressed in human hair follicles, and its mutations lead to hereditary hypotrichosis simplex, which is a rare autosomal dominant form of hair loss [56].

Secretory reticular fibroblasts in the reticular dermis exhibit a wide range of functional variation, which is thought to be caused by changes in anatomical location, tissue, or activity. Interestingly, secretory reticular fibroblasts have a strong expression signature for genes involved in ECM formation, wound healing, and angiogenesis, such as collagens (COL1A1, COL1A2), elastin (ELN), fibronectin (FN1), profibrillin (FBN1), microfibril-associated proteins (MFAP4, MFAP5), and lysyl oxidase (LOX). [55]. Indeed, some of those markers have been indicated in Table 1. The involvement of these markers in various physiological processes highlights their significance in maintaining dermal integrity and facilitating tissue repair. Among those, however, only one, Tetraspanin 8 (TSPAN8), was found to be a cell surface protein, which can be used for sorting and characterizing live secretory reticular fibroblast cells. TSPAN8 plays a role in cell motility and invasion, contributing to processes such as wound healing and angiogenesis [57].

Fibroblasts classified as pro-inflammatory express genes encoding proteins involved in inflammatory response, cell chemotaxis, or negative regulation of cell proliferation, such as C-C motif chemokine 19 (CCL19), Apolipoprotein E (APOE), C-X-C motif chemokine 2 (CXCL2), C-X-C motif chemokine 3 (CXCL3), and EGF-containing fibulin-like extracellular matrix protein 1 (EFEMP1). Those markers were also described in the papers of Solé-Boldo L. et al. [26], Riedl JA et al. [27], and Shim J et al. [16], as shown in Table 1, but none of the identified markers can be classified as surface markers.

The last subpopulation identified among normal human fibroblasts during scNGS are cells named mesenchymal fibroblasts, which are localized in the reticular dermis and express a wide range of protein-encoding genes such as Asporin (ASPN), Periostin (POSTN), Glypican-3 (GPC3), Tenascin-N (TNN), and secreted frizzled-related protein 1 (SFRP1). Among those, only Glypican-3 is a surface heparan sulfate proteoglycan and can potentially be used for fibroblast isolation via cell sorting. Interestingly, GPC3 was found to be up-regulated in the subgroups of cancer-associated fibroblasts in advanced gastric cancer and correlates with poor patient prognosis [58]. Still, the surface markers mentioned above for the selected subpopulation of fibroblasts are not specific and cannot be solely used for their selection. However, if combined with other common markers of fibroblasts, described later in the Discussion section, it might be possible to isolate these subpopulations. Therefore, the isolation and characterization of these subgroups should be further investigated.

During the aging process, skin undergoes numerous structural, physiological, and bio-chemical alterations driven by the cumulative effects of extrinsic factors, such as ultraviolet radiation and pollutants, in conjunction with limited regenerative potential [59]. Single-cell analysis reveals that fibroblasts exhibit the highest degree of transcriptional age-related variability among skin cells. This variability is characterized by the up-regulation of pro-inflammatory cytokines and extracellular matrix remodeling proteins such as MMP2, and the down-regulation of genes involved in cellular proliferation [40]. Additionally, there are quantitative changes, notably the decreased abundance of papillary and mesenchymal fibroblast subpopulations in skin samples from elderly individuals [26,41]. A study by Ahlers et al. [41] identified a unique Dermal Sheath Fibroblast subpopulation localized in the dermal sheath cup of the hair bulb. This subpopulation, present in skin samples from young individuals, exhibits stem cell characteristics, including adipogenic and chondrogenic differentiation capacity and high proliferative potential. Additionally, through cytokine secretion, particularly Activin A, it mediates paracrine effects on other major skin cell types, contributing to a youthful skin phenotype. The reduction in this subpopulation may elucidate age-related changes across fibroblast subtypes and the loss of fibroblast priming.

Aside from normal skin, fibroblasts from several skin diseases were studied during scNGS (Table 1, Table 2, Table 3, Table 4 and Table 5). Normal wound healing requires controlled fibroblast proliferation, migration, matrix deposition, and contraction. Dysregulation in these processes can result in excessive collagen formation, as shown in keloids, which grow beyond the initial wound edges [44]. Characteristic features of keloids include uncontrolled fibroblast proliferation and excessive extracellular matrix (ECM) production [60].

Fibroblasts found in keloids highly express α-SMA, suggesting their myofibroblast phenotype. Keloid fibroblasts exhibit greater amounts of TGF-β1 receptors, indicating that abnormal TGF-β1 responses, which influence collagenous ECM production and deposition, contribute to keloid development [44]. Interestingly, the mesenchymal subpopulation of fibroblasts was higher in keloid compared to normal scar tissue, but the proportions of secretory papillary, secretory reticular, and pro-inflammatory subpopulations were lower. In these cells, elevated expression of membrane proteins such as syndecan-1 (SDC1), ADAM Metallopeptidase Domain 12 (ADAM12), and fibroblast growth factor-inducible immediate-early response protein 14 (TNFRSF12A), known as CD266, has been found [19]. As a result, in the future, these markers may be utilized to identify this specific subpopulation and create new treatment alternatives, as keloids are now difficult to treat due to their high chance of recurrence and poor clinical results [16,28].

Fibroblasts in atopic dermatitis exhibit a variety of genes, including those involved in immune modulation and pathological alterations within the skin; nevertheless, the findings are inconclusive and do not identify key fibroblast subpopulations responsible for the disease. In the paper by Alkon et al. [29], the CD74 protein is found in so-called inflammatory fibroblasts. CD74 is a cell surface receptor for the cytokine macrophage migration inhibitory factor and plays a crucial role in antigen presentation by stabilizing MHC class II molecules on the surface of antigen-presenting cells. It is implicated in inflammatory processes and may influence the pathogenesis of various inflammatory diseases [33,61]. Although interesting, this discovery was not confirmed by He et al. [20] and Zhang et al. [30] (Table 2), who described several proteins expressed in fibroblast subsets of atopic dermatitis. Among those proteins, only Vascular Cell Adhesion Molecule 1 (VCAM1), Endothelin Receptor Type A (EDNRA), and Interferon-Induced Transmembrane Proteins 2 and 3 (IFITM2/IFITM3) were identified as surface proteins, and no information was provided about CD74 expression. Interestingly, CD74 protein was found in subsets of fibroblasts in prurigo nodularis, a chronic skin condition characterized by very itchy, firm lumps [31], and in a subset of antigen-presenting fibroblasts in lupus erythematosus, a chronic disease that causes inflammation in connective tissues (Table 3) [32].

During analysis, we observed inconsistency in fibroblast nomenclature. For example, in prurigo nodularis, either different subset of fibroblasts were counted [31], or they were classified into papillary and reticular secretory cells [29]. In those articles, APCDD1 and Integral Membrane Protein 2A (ITM2A) cell surface proteins are common in some of the described fibroblast subsets; nevertheless, the additionally detected non-surface proteins in those cells do not allow for indication of whether the cells are part of the same group or different groups.

Analyses of fibroblast subsets in pre-cancerous skin conditions and skin malignancies have also revealed a broad spectrum of fibroblast heterogeneity (Table 4). Actinic keratosis (AK), a pre-cancerous skin disease characterized by the proliferation of atypical keratinocytes, has been linked to interactions between AK keratinocytes and secretory papillary fibroblasts, which may be implicated in the development and progression of atypical keratinocytes [34]. It would be extremely interesting to analyze the behavior of sorted fibroblasts and their interaction with AK keratinocytes, especially given the novel, easier protocols that allow for 3D skin model cultures [62]. However, no conclusive surface proteins have been described as characteristic of this fibroblast subset. According to our findings, no significant data involving fibroblast subsets were found for basal cell carcinoma [35,36,37] or squamous cell carcinoma [38,39]. Separate subsets of fibroblasts were detected in various disorders; however, the identified surface proteins did not overlap in the articles reviewed. This makes it difficult to determine whether these subsets are the same or distinct groups of fibroblasts, indicating the need for further research.

### 4.2. Fibroblasts’ Markers Utilized in Antibody-Based Techniques

Another approach for identifying fibroblast subpopulations involves analyzing sorted fibroblasts, primarily through FACS. Researchers often study fibroblasts based on their location within the dermis (Table 6). In our investigation, Thy-1 was the most frequently used surface marker for sorting viable fibroblasts (Table 6). CD90, also known as Thy-1, is a highly conserved protein that connects to the cellular membrane via a glycophosphatidylinositol anchor. It is involved in a variety of physiological processes, including cell migration, differentiation, apoptosis, and cell division. Recent studies show that it plays a crucial role in cancer formation by regulating the WNT/β-catenin pathway and epithelial–mesenchymal transition [48]. Interestingly, two cell surface markers, CD90 and FAP, can be used to separate papillary and reticular fibroblasts from human skin using FACS. Fibroblast Activation Protein (FAP) is a type II transmembrane serine protease belonging to the prolyl peptidase family. Elevated FAP expression has been identified in activated fibroblasts and stromal fibroblast-like cells in numerous human diseases and cancers [50,63]. Additionally, FAP’s role in matrix remodeling and its overexpression in the tumor microenvironment highlight its potential as a therapeutic target in cancer treatment.

Interestingly, FAP and CD90 can only be used to separate fibroblast subpopulations directly from the skin but not to differentiate reticular and papillary fibroblasts in mixed in vitro cell cultures due to changes in cell surface marker expression during two-dimensional in vitro culture [7]. Fibroblasts from the papillary dermis promote the establishment of a properly stratified epidermis in three-dimensional organotypic culture more efficiently than fibroblasts from the reticular dermis [42]. Additionally, fibroblasts from the deep dermis proliferate more slowly in culture compared to those obtained from upper dermal regions [45]. Despite this, cell sorting of viable fibroblasts enables in vitro research of lineage-specific properties in both healthy and diseased conditions, as fibroblast subsets isolated from the dermis retain their distinct function when cultured separately, despite changes in cell surface marker expression [24]. According to functional analysis and expression profiling, the FAP+CD90- phenotype of papillary fibroblasts in the skin is characterized by high proliferation rates and the absence of adipogenic potential [24]. These fibroblasts are primarily found in the upper dermis, though not exclusively. Conversely, FAP+CD90+ and FAP-CD90+ fibroblasts belong to the reticular lineage of the lower dermal compartments. Reticular fibroblasts can also be present in the papillary dermis, but their numbers are significantly lower [7]. Although reticular fibroblasts have a lower proliferation rate, they are noted for their propensity to undergo adipogenesis [24]. Notably, despite their predominant locations in the upper and lower dermis, papillary and reticular fibroblasts are not spatially constrained, and there is an opposing gradient of these two fibroblast lineages from the skin’s surface to the hypodermis [7].

In our analysis, the second most frequently used surface marker for the fibroblast subset during FACS was CD26 (Table 6). CD26, also known as dipeptidyl peptidase-IV (DPP4) or adenosine deaminase complexing protein 2, is a cell surface glycoprotein that regulates the inflammatory response. CD26+ fibroblasts characterize a population of activated fibroblasts believed to contribute to skin fibrosis, presumably via the TGFβ pathway [64]. Murine studies have shown that CD26 (DPP4) is a marker of Engrailed-1-positive cells, and its inhibition reduces wound scarring [25]. Additionally, murine studies indicate that CD26 identifies an upper dermal lineage during early mouse development, whereas in adult mice, the majority of CD26 is associated with the reticular lineage [65]. Although several cell surface markers observed in the mouse dermis are not conserved in humans, CD26 expression was confirmed by Philippeos et al., who described CD26+ fibroblasts in a significant proportion of the adult human dermis but did not detect them in the uppermost (papillary) fibroblasts [42]. Interestingly, Korosec et al. identified CD26 in CD90+ reticular fibroblasts within both the superficial and deep dermis, as well as in a subset of papillary fibroblasts in the superficial dermis [7]. In human wounds, the number of CD26+ fibroblasts increases significantly, implicating them as primary cells in the wound-healing process [43]. Additionally, higher expression of CD26 and FAP fibroblasts was observed in keloids [46]. Furthermore, the skin of both young and elderly individuals contains similar amounts of CD26+ fibroblasts, suggesting that the age-associated loss of collagen production is not due to a selective decrease in CD26+ fibroblasts [43]. Tabib et al. also identified CD26+ fibroblasts as belonging to a papillary lineage and found that two primary fibroblast populations can be differentiated by their expression of SFRP2/DPP4 or FMO1/LSP1 markers [25].

The SFRP2+ fibroblasts were dispersed within the collagen bundles, appearing small and elongated. In contrast, FMO1+ fibroblasts were found in both interstitial and perivascular regions and were larger in size [25]. Unfortunately, among those markers, only DPP4 may be regarded as a surface protein and can be used for viable cell sorting. Interestingly, both of these populations also expressed CD34 surface protein. The presence of this marker was also confirmed by Worthen et al. [43]. This study showed that utilizing both CD26 and CD90 cell surface glycoproteins enables the identification of a specialized fibroblast population that expresses COL1A1, a fibroblast-specific protein, at very high levels [43]. In these fibroblasts, CD34+/CD26+/CD90+ high COL1A1 gene expression was observed, whereas CD90- cell populations (CD26-/CD90- and CD26+/CD90-) exhibited minimal to no COL1A1 expression [25].

CD34+ fibroblasts are intrinsic stromal components of nearly all organs, playing critical roles in matrix production, antigen presentation, and tumor-associated stromal remodeling. In invasive breast carcinoma, the loss of CD34+ fibroblasts is accompanied by the emergence of α-SMA+ myofibroblasts, also known as cancer-associated fibroblasts (CAFs) [66]. Although further studies are needed, this finding may have diagnostic relevance and therapeutic implications.

Based on our analysis, CD36 was the third most commonly used marker for FACS-based viable cell sorting (Table 6). CD36, a transmembrane glycoprotein, is expressed in a wide range of cell types and plays a crucial role in various physiological processes, including inflammation, angiogenesis, and fatty acid metabolism [67]. While CD26 was expressed in both the papillary and reticular dermis, with higher levels reported in the upper layer, CD36 was reported to be mostly identified in the deeper dermis, [7]. In fibroblasts, CD36 expression is associated with their adipogenic potential. Fibroblasts isolated primarily from the papillary dermis (CD90+CD36–) did not differentiate into adipocytes, whereas fibroblasts isolated primarily from the upper and lower reticular dermis (CD90+CD36+) demonstrated a robust capacity for adipogenic differentiation [7].

Fibroblasts from the deep dermis were previously considered to possess higher fibrogenic potential, whereas those from the superficial dermis were thought to be non-fibrogenic [46]. However, this may not be entirely accurate, as a positive correlation has been found between CD39+ fibroblasts, which were previously believed to be a subset of non-fibrogenic fibroblasts with papillary-like biological activity localized in the upper dermis, and the severity of hypertrophic scars (HTSs). Furthermore, targeted suppression of CD39+ fibroblasts significantly reduces scarring and skin fibrosis in vivo, introducing a promising new approach for antiscarring therapeutics aimed at specific fibroblast subtypes [46]. According to our data (Table 6), CD39 was the next most commonly employed marker for viable cell sorting using FACS. CD39 is a surface ectonucleotidase involved in the hydrolysis of ATP in the extracellular space and serves as a conserved marker in both human and mouse papillary fibroblasts [46].

CD90+CD39+ fibroblasts in the upper dermis are more effective in promoting the formation of a multilayered epithelium in three-dimensional organotypic culture compared to CD90+CD39- and CD90+CD36+ fibroblasts, which are found in the mid and deep dermis [42]. Upper dermal fibroblasts (CD90+CD39+) exhibit an anti-inflammatory phenotype when activated by IFN-γ, unlike those in the lower dermis/hypodermis (CD90+CD36+) when cultured in vitro [42]. Additionally, CD39+ fibroblasts produce high levels of IL-11, a cytokine elevated in hypertrophic scars, highlighting the importance of identifying these lineages in future studies [46]. After a single passage in cell culture, CD39 expression was lost in isolated fibroblasts, whereas CD90 and CD36 expression was retained. Even though CD90+CD39+ and CD90+CD36+ cells exhibit distinct morphologies in vitro [42]. Thus, isolating viable cells and analyzing specific fibroblast subtypes may facilitate the detailed examination of their unique characteristics.

## 5. Conclusions

To conclude, the majority of research on fibroblast activities has been conducted using non-sorted cell populations, resulting in a limited understanding of their diversity and multifunctionality. Future research, particularly utilizing single-cell analysis and FACS, should focus on identifying surface markers that enable the in vitro culture and differentiation of fibroblast subgroups, allowing for detailed analysis of their features and responses to various stimuli. Interestingly, despite the rapid loss of key subpopulation marker expression during in vitro culture, the functional characteristics of upper and lower dermal fibroblast subpopulations persisted following in vitro expansion, at least during the initial passages [42]. While the number of mediators known to activate fibroblasts continues to grow, our understanding of molecules that inhibit their function remains extremely limited.

**Figure 2:**
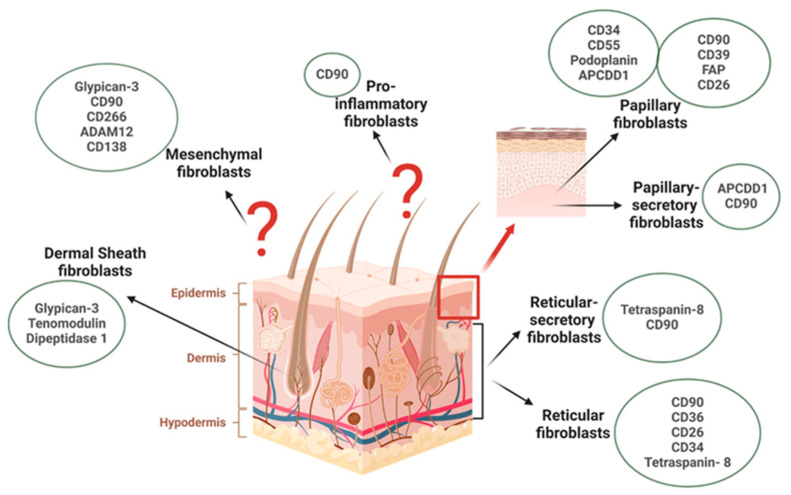
A graphical summary of different fibroblast subtypes in human skin.

Through the analyses, four major subpopulations of skin fibroblasts were consistently identified (Figure 2). Papillary fibroblasts were characterized by the expression of CD26, CD34, CD39, CD55, CD90, Fibroblast Activation Protein (FAP), Podoplanin, and APCDD1, with a subgroup of papillary secretory fibroblasts distinguished by CD90 and APCDD1. Reticular fibroblasts were identified by the expression of CD26, CD34, CD36, CD90, and Tetraspanin-8, with a subgroup of reticular secretory fibroblasts characterized by CD90 and Tetraspanin-8, located in the respective layers of the dermis. Additionally, mesenchymal fibroblasts, marked by the expression of CD90, CD138, CD266, ADAM12, and Glypican-3, and pro-inflammatory fibroblasts, characterized solely by CD90 surface markers, were evenly dispersed across the skin layers. It is important to note that pro-inflammatory fibroblasts cannot be selected via CD90 alone. Therefore, future studies are needed to identify additional markers.

Research on skin aging revealed a distinct population of Dermal Sheath Fibroblasts, characterized by the expression of Glypican-3, Tenomodulin, and Dipeptidase 1, specifically located in the hair bulb sheath of skin samples from young patients. While analyses from healthy samples were consistent, studies on diseased skin revealed a variety of unique disease-associated fibroblast subtypes lacking common surface markers. While CD74 is overexpressed in skin inflammatory diseases and Fibroblast Activation Protein (FAP) is overexpressed in cutaneous cancers, implying a potential role of these markers in disease pathogenesis, isolating cells based solely on a single marker remains impossible.

Our analyses highlight the most common surface markers, providing a basis for further research and additional studies comparing these subpopulations using a pre-set panel of markers are necessary. Consequently, with a better understanding of fibroblasts’ biology and their role in the immune response, novel therapeutic options for the inhibition of deleterious fibroblasts are likely to emerge.

## Figures and Tables

**Table 1:** Main positive surface and non-surface markers of fibroblast subpopulations identified after single-cell sequencing analyses in normal skin and keloid tissues. The intracellular markers are labeled in red, the extracellular matrix markers are labeled in green, and the secreted markers are labeled in blue.

Ref.	Tissue and Site/Disease	Fibroblasts Population	Main Positive Surface Markers	Main Positive Non-Surface Markers
[25]	Normal skin (Dorsal Forearm)	Papillary/[SFRP2/DPP4] fibroblasts	CD26, CD34, CD55	NKD2, SFRP2, PCOLCE, WIF1
		Reticular/[FMO1/LSP1] fibroblasts	CD34	FMO1, LSP1, MYOC, IGFBP3
[26]	Normal skin (Inguinoiliac Region)	Papillary-secretory fibroblasts	Protein APCDD1	ID1, WIF1, COL18A1, PTGDS
		Reticular-secretory fibroblasts	Tspan-8	CTHRC1, MFAP5, WISP2, SLPI
		Mesenchymal Fibroblasts	Glypican-3	TNN, POSTN, SFRP1, ASPN, APOE
		Pro-inflammatory fibroblasts	-	CCL19, CXCL2, CXCL3, EFEMP1
[27]	Allografted RDBE skin (Flank, Upper Thigh, Forearm)	Papillary-secretory fibroblasts	Protein APCDD1	ID1, WIF1, COL18A1, PTGDS
		Reticular-secretory fibroblasts	Tspan-8	CTHRC1, MFAP5, WISP2, SLPI
		Mesenchymal fibroblasts	Glypican-3	TNN, POSTN, SFRP1, ASPN, APOE
		Pro-inflammatory fibroblasts	-	CCL19, CXCL2, CXCL3, EFEMP1
[16]	Normal skin vs. Keloid (Inguinoiliac Region, Back, Ear)	Papillary-secretory fibroblasts	Protein APCDD1	WIF1
		Reticular-secretory fibroblasts	-	WISP2, SLPI
		Mesenchymal fibroblasts	-	POSTN, ASPN
		Pro-inflammatory fibroblasts	-	CCL19, APOE
[19]	Normal skin vs. Keloid (Chest, Back)	Papillary-secretory fibroblasts	CD90	COL13A1, COL18A1, COL23A1
		Reticular-secretory fibroblasts	CD90	MDK, COL1A1, SPARC, COL5A2, APOE
		Mesenchymal fibroblasts	CD90, CD266, ADAM 12, CD138	NREP, COL11A1, COMP, POSTN
		Pro-inflammatory fibroblasts	CD90	COL1A1, COL1A2, COL3A1, SFRP4, SPARC
[28]	Keloid–(Chest)	Fibroblasts	CD140a	RGS2, REL, DCN, POSTN, ABCA8, WISP

**Table 2:** Main positive surface and non-surface markers of fibroblast subpopulations identified after single-cell sequencing analyses in inflammatory and autoimmune skin diseases. The intracellular markers are labeled in red, the extracellular matrix markers are labeled in green, and the secreted markers are labeled in blue.

Ref.	Tissue and Site/Disease	Fibroblasts Population	Main Positive Surface Markers	Main Positive Non-Surface Markers
[29]	Atopic dermatitis–(Trunk, Arm, Leg)	Inflammatory fibroblasts	CD74	EFEMP1, APOE, CCL19, CCL2
[20]	Normal skin and atopic dermatitis (Extremities)	Fibroblasts	Endothelin-1 receptor, ABCA6	COL1A1, DCN, APOE, APOD, C3
		Fibroblasts	ABCA6	COL1A1, FBN1, MFAP5, DCN
		Fibroblasts	-	COL1A1, POSTN, COL11A1, LAMC3, DCN
		Inflammatory fibroblasts	VCAM-1	COL6A5, COL18A1, POSTN, CCL2, CCL19, IL32, TNC
[30]	Atopic dermatitis–(Arm, Leg, Back)	Fibroblasts	IFITM-2/IFITM-3	MT1X, LGALS3, POSTN, COL6A5, COL6A6, IGFBP6, TNC
	Psoriasis–(Leg, Arm)	Fibroblasts	-	LGALS3, HSPA1B
[22]	Psoriasis (Lower back, Buttocks)	Fibroblasts	-	PI16, CCN5
		Fibroblasts	-	COMP
		Fibroblasts	-	APOE, CXCL3
		Fibroblasts	-	APOE, CCL19
		Fibroblasts	-	APOE, C7
		Fibroblasts	Dipeptidase 1	COL11A1
		Fibroblasts	-	COCH, ASPN
		Fibroblasts	-	ANGPTL7
		Inflammatory fibroblasts	CD120a, CD120b, IL17-RA/IL17-RC	WNT5A, IL24

**Table 3:** Main positive surface and non-surface markers of fibroblast subpopulations identified after single-cell sequencing analyses in inflammatory and autoimmune skin diseases (cont’d). The intracellular markers are labeled in red, the extracellular matrix markers are labeled in green, and the secreted markers are labeled in blue.

Ref.	Tissue and Site/Disease	Fibroblasts Population	Main Positive Surface Markers	Main Positive Non-Surface Markers
[31]	Prurigo nodularis (Not specified)	Fibroblasts	CD90	NKD2, POSTN, WNT5A, DIO2
		Fibroblasts	CD74	PTGDS, CCL19, C3
		Fibroblasts	-	SFRP2, FBN1, TNXB
		Fibroblasts	Integral membrane protein 2A	PI16, CXCL12
		Fibroblasts	-	SFRP1, TIMP3, ASPN
		Fibroblasts	Protein APCDD1	-
		Fibroblasts	-	TAGLN, FOXS1, APOD
[29]	Prurigo nodularis (Trunk, Arm, Leg)	Papillary-secretory fibroblasts	Protein APCDD1	COL18A1, COL13A1, COL23A1
		Reticular-secretory fibroblasts	CD26	MFAP5, MMP2, FBN1, ELN, CCN5, SLPI
		Inflammatory fibroblasts	Integral membrane protein 2A	SPSB1, EFEMP1, APOE, CXCL2, CXCL3
		Proliferating fibroblasts	-	MKI67
[32]	Lupus erythematosus (Face, Neck, Forehead, Arm)	Fibroblasts	-	PI16, DCN, COL1A1, COL1A2, SFRP2, FBLN1
		Fibroblasts	Gap junction alpha-4 protein	RGS5, RGS16, ID4, NR2F2, COL1A1, DCN, CCL2
		Fibroblasts	Scn7a, CD49f, Claudin-1	DCN, SFRP4, CYP1B1, PTGDS, APOD
		Fibroblasts	-	COL1A1, DCN, STMN1, CENPF, PTTG1
		Inflammatory fibroblasts	-	PLIN2, COL1A1, DCN, CXCL1, CXCL2, CXCL3
		Antigen- presenting fibroblasts	CD74, HLA-DRA1/DRB1; HLA-DQB1	SRGN, TYROBP, COL1A1, DCN, C1QA, CXCL12
		Antigen- presenting fibroblasts	CD74, HLA-DRA1/DRB1, TM4SF1 Protein	RGS5, CITED4, ACKR1, COL1A1, DCN, STC1
[33]	Hidradenitis suppurativa–(Trunk)	Inflammatory fibroblasts	-	POSTN, IL1B, IL6, IL11, IL24
[23]	Vitiligo–(Extremities, Head, Back)	Inflammatory fibroblasts	-	TWIST2, DCN, SFRP2, CCL19

**Table 4:** Main positive surface and non-surface markers of fibroblast subpopulations obtained from single-cell sequencing analyses in skin diseases involving dysplasia and malignancies. The intracellular markers are labeled in red, the extracellular matrix markers are labeled in green, and the secreted markers are labeled in blue.

Ref.	Tissue and Site/Disease	Fibroblasts Population	Main Positive Surface Markers	Main Positive Non-Surface Markers
[34]	Normal skin and Actinic keratosis (Face)	Papillary-secretory fibroblasts	Protein APCDD1	COL18A1, PTGDS
		Reticular-secretory fibroblasts	Tspan-8	CTHRC1, MFAP5, WISP2, SLPI
		Mesenchymal fibroblasts	Glypican-3	POSTN, SFRP1, ASPN
		Pro-inflammatory fibroblasts	-	CCL19, APOE, EFEMP1
[35]	Basal Cell Carcinoma (Leg, Elbow, Head)	CAFs 1	SLIT and NTRK-like protein 6	COCH, OGN, ENPP2, CYP1B1
		CAFs 2	HLA-DRA, CXCR-4	SRGN, CREM, BTG1
		CAFs 3	-	APOD, C3, CFD, CXCL14, CCL19
		CAFs 4	-	IGFB3, IER3, MMP1, MMP3, SAT1
		CAFs 5	FAP	TAGLN, POSTN, COMP, ACTA2, MMP11
[36]	Basal Cell Carcinoma (Leg, Head)	CAFs	CD34, RGMA, SCARA5	FBLN1, CXCL12, GPX3
		CAFs	CD74	CCL5
		Quiescent CAFs	Notch3	SOX9, SOX10, EPAS1, IL6, PDGFA
		ECM-remodelling CAFs	FAP, Lrrc15 protein	TAGLM, POSTN, COL1A1, MMP2, ACTA2
[37]	Normal skin vs Basal Cell Carcinoma (Face)	Papillary Fibroblasts	-	PTGDS
		Reticular Fibroblasts	-	SFRP2, WISP2
		Fibroblasts proximal to blood vessels	-	APOE, APOD
		POSTN+ Fibroblasts	-	POSTN
[38]	Normal skin vs Squaomus cell carcinoma Healthy–(Inguinal region, Head) Squamous Cell Carcinoma (Head)	Papillary-secretory fibroblasts	Protein APCDD1	COL18A1, DIO2, PTGDS, FMOD, LTBP1
		Reticular-secretory fibroblasts	-	PI16, FBLN1, MFAP5, SLPI, WISP2, IGFBP6
		Mesenchymal Fibroblasts	-	TAGLN, POSTN, TPM2, MMP11, FN1
		Pro-Inflammatory fibroblasts	-	CCL19, CXCL3, APOE, CXCL1, APOD
		Inflammatory CAFs	-	MGP, JUND, JUN, HSP90AA1, XBP1, IGF1
		Myofibroblast CAFs	-	TAGLN, COL11A1, SULF1, TDO2, MMP11, WNT5A
[39]	Normal skin vs. Squamous cell carcinoma (Head, Foot)	Myofibroblast CAFs	-	RGS5, COL6A2, COL1A1, COL1A2, DCN
		Inflammatory CAFs	IL7-R	RGS5, COL6A2, COL1A1, COL1A2, DCN
		CAFs	IL7-R	COL6A2, COL1A1, COL1A2, DCN, IL1B, CXCL1, IL6, CXCL3, CXCL5, CXCL6, CXCL8, CXCL13, CXCL14

**Table 5:** Main positive surface and non-surface markers of fibroblast subpopulations obtained from single-cell sequencing analyses in skin aging. The intracellular markers are labeled in red, the extracellular matrix markers are labeled in green, and the secreted markers are labeled in blue.

Ref.	Tissue and Site/Disease	Fibroblasts Population	Main Positive Surface Markers	Main Positive Non-Surface Markers
[40]	Skin aging (Upper Eyelid)	Reticular Fibroblasts	-	MFAP5, MGP
		Reticular Fibroblasts	-	MFAP5, MGP
		Papillary Fibroblasts	Podoplanin	PTGDS
[26]	Skin aging (Inguinoiliac region)	Secretory-Reticular Fibroblasts	Tetraspanin-8	CTHRC1, MFAP5, WISP2, SLPI
		Pro-Inflammatory Fibroblasts	-	APOE, CXCL1, CXCL2, CXCL3, EFEMP1, CCL19
		Secretory-Papillary Fibroblasts	Protein APCDD1	ID1, WIF1, COL18A1, PTGDS
		Mesenchymal Fibroblasts	Glypican-3	TNN, POSTN, SFRP1, ASPN
		Dermal-Papilla associated Fibroblasts	-	TNN, CRABP1
[41]	Skin aging (Outer Forearm)	Reticular Fibroblasts	Tetraspanin-8	CTHRC1, MFAP5, WISP2, SLPI
		Papillary Fibroblasts	Protein APCDD1	ID1, WIF1, COL18A1, PTGDS
		Inflammatory Fibroblasts	-	CCL19, APOE, CXCL2, CXCL3, EFEMP1
		Mesenchymal Fibroblasts	Glypican-3	TNN, POSTN, SFRP1, ASPN
		Dermal Sheath Fibroblasts	Dipeptidase 1, Tenomodulin, Glypican-3	PMEPA1, TAGLN, MEF2C, MYL4, POSTN, COL11A1, SPARC , WFDC1

**Table 6:** Main positive surface and non-surface markers of fibroblast subpopulations obtained using FACS or other antibody-based analyses. The intracellular markers are labeled in red, the extracellular matrix markers are labeled in green, and the secreted markers are labeled in blue.

Ref.	Tissue and Site/Disease	Fibroblasts Population	Main Negative Surface Markers	Main Positive Surface Markers	Main Positive Non-Surface Markers
[42]	Normal skin (Not specified)	Papillary fibroblasts	CD26, CD31, CD45, E-cad	CD39, CD90	LEF1, VIM, COL6A5, WNT5A, RSPO1
		Reticular fibroblasts	CD31, CD45, E-cad	CD26, CD36, CD90	MFAP5, PRG4, VIM, DCN, LUM
[7]	Normal skin (Abdomen, Chest)	Papillary fibroblasts	CD31, CD45, E-cad, CD106, CD235, CD90	CD26, CD39, PDPN, FAP	NTN1
		Reticular fibroblasts	CD31, CD45, E-cad, CD106, CD235, FAP	CD36, CD90	αSMA, MGP
[24]	Normal skin (Abdomen, Chest)	Papillary fibroblasts	CD31, CD45, E-cad, CD106, CD235, CD90	FAP	-
		Reticular fibroblasts	CD31, CD45, E-cad, CD106, CD235, FAP	CD90	-
[43]	Normal and wounded skin (Buttocks)	CD26-/CD90-	CD26, CD31, CD45, CD235, CD90	-	-
		CD26+/CD90-	CD31, CD45, CD235, CD90	CD26	-
		CD26-/CD90+	CD26, CD31, CD45, CD235	CD90	COL1A1
		CD26+/CD90+	CD31, CD45, CD235	CD26, CD34, CD90	COL1A1
[44]	Keloid Palmoplantar region	Myofibroblasts	-	TGFB1R	αSMA
		Fibroblasts	-	TGFB1R	-
[45]	Normal skin and HTS Normal–(Trunk) HTS–(Hand, Chest)	HTS Fibroblasts	-	-	αSMA, DCN, Versican, TGF-β1
[46]	Normal skin and HTS Normal–(Trunk) HTS–(Face, Neck, Chest, Back, Arm)	HTS fibroblasts	CD26, CD31, CD36, CD45, E-cad, CD49f, FAP	CD39, CD90	NLRP3, IL11, CXCL1
		Fibroblasts	CD45, E-cad, CD49f	CD90, FAP	-
		Fibroblasts	CD45, E-cad, CD49f	CD26, CD90	-
		Fibroblasts	CD31, CD45, E-cad, CD49f, CD39	CD36, CD90, CRHR1, SYNGR2	CBLN4

## Data Availability

All relevant data are included within the manuscript. The raw data are available on request from the corresponding author.
